# Tumor-associated macrophages promote ferroptosis resistance in glioblastoma by stimulating iron-loaded extracellular vesicle release

**DOI:** 10.1007/s11060-026-05556-w

**Published:** 2026-04-13

**Authors:** Aurosman Pappus Sahu, Kondaiah Palsa, Ganesh Shenoy, Becky Slagle-Webb, James R. Connor

**Affiliations:** 1 Department of Neurosurgery, Penn State College of Medicine, Hershey, PA 17033 USA; 2 Department of Medicine, Johns Hopkins University School of Medicine, Baltimore, MD USA

**Keywords:** Ferroptosis, Glioblastoma, Tumor-associated macrophages, Iron metabolism, Extracellular vesicles, Tumor microenvironment

## Abstract

**Purpose:**

Glioblastoma (GBM) cells have a higher iron requirement than normal cells and therefore upregulate iron uptake. However, elevated intracellular iron levels can render cancer cells susceptible to ferroptosis, a non-apoptotic, iron-dependent form of cell death. Tumor-associated macrophages (TAMs) are the most abundant non-tumor cell type in GBM and play a pivotal role in GBM iron homeostasis. Although the role of TAMs in regulating iron availability in the TME is well established, their role in regulating ferroptosis in the GBM cells remains unexplored.

**Methods:**

A transwell coculture system was used involving GL261 cells and bone marrow–derived macrophages (BMDMs) from C57BL/6J mice to study cancer cell- TAM interaction in GBM. In vitro analyses including immunoblot, cell death assay, lipid peroxidation assay and labile iron pool measurements were done to examine the iron and ferroptotic status of GL261 cells. Extracellular Vesicles (EVs) were isolated by ultracentrifugation. Pharmacological inhibition of EVs was done to confirm the mechanism of action of TAM-induced ferroptosis resistance.

**Results:**

TAMs rendered GL261 cells more resistant to RSL3-induced ferroptotic stress. Mechanistically, TAMs reduced Gl261 cellular iron levels by increasing the release of H-ferritin–bound iron via CD63-positive EVs, with a male sex bias. TAM-secreted TNF-α played a key role in promoting this ferritin-bound iron release. Treatment with GW4869, a potent inhibitor of EV formation, in the presence of hepcidin resensitized TAM-cocultured GL261 cells to RSL3-induced ferroptosis.

**Conclusions:**

These findings indicate that TAMs protect GBM cells from ferroptotic stress by inducing EV-mediated ferritin-bound iron release from the GBM cells through TNF-α signaling.

**Supplementary Information:**

The online version contains supplementary material available at 10.1007/s11060-026-05556-w.

## Introduction

Glioblastoma (GBM) is one of the most difficult-to-treat cancers, with a 5-year survival rate of around 6% [1]. It is a solid tumor of astrocytic origin and characterized by a unique tumor microenvironment (TME) consisting of tumor cells, brain microglia, and infiltrating immune cells [2]. The role of iron in tumor progression has been understudied, especially in the context of GBM [3, 4]. Iron plays a vital role in human physiology, given that it is actively involved in oxygen transport. Iron is essential for several cellular processes, such as mitochondrial ATP production, DNA synthesis, and cell proliferation [5–7]. Cancer cells have a higher iron requirement than normal cells; therefore, they upregulate the expression of the transferrin receptor (TFRC), a protein involved in iron uptake. However, due to the high iron uptake in cancer cells, they are susceptible to ferroptosis, an iron-mediated form of cell death. Ferroptosis is characterized by increased cellular free iron and lipid peroxidation [8]. Lipid peroxidation occurs as a result of the Fenton reaction, where ferrous iron (Fe^2+^) reacts with H_2_O_2_, to produce hydroxyl radicals. These hydroxyl radicals further react with phospholipid hydroperoxides to form phospholipid hydroperoxide radicals, which can cause membrane damage [9, 10]. However, cancer cells have developed multiple strategies to suppress ferroptotic stress, including boosting antioxidant defenses, reducing iron overload, and modulating lipid metabolism. For example, breast and ovarian cancer cells have been shown to resist ferroptotic stress by inducing iron release, thereby limiting the iron available for the Fenton reaction [11]. Yet the mechanisms involved in conferring GBM cells’ resistance to ferroptotic stress remain poorly understood.

Tumor Associated Macrophages (TAMs) comprise up to 50% of the total tumor volume in GBM and are one of the most abundant cell populations in TME. The role of TAMs in tumorigenesis has been extensively documented, particularly in terms of cytokine secretion, immune cell recruitment, and altering the metabolic landscape of the TME [12]. Notably, in iron metabolism, TAMs play a significant role in maintaining iron homeostasis in the TME. In several tumor types, it has been reported that TAMs can affect the ferroptotic state of the tumor cells. This is because TAMs regulate iron availability to cancer cells through direct iron delivery, as well as by regulating tumor iron retention and release via different signaling pathways [13]. The only known mechanism by which TAMs protect tumor cells from ferroptosis is by facilitating ferroportin (FPN)-mediated iron export from cancer cells, resulting in ferroptosis suppression [14]. However, in several tumors, such as GBM, the proinflammatory nature of the TME leads to inhibition of FPN-mediated iron export due to elevated hepcidin expression [15]. Hence, there may be alternate ferroptosis suppression pathways active during inflammation that are independent of the FPN/Hepcidin regulation. Overall, the role of TAMs in regulating ferroptosis in the GBM tumor microenvironment remains unexplored.

In this current study, we use primary macrophages from C57BL/6J mice and syngeneic GL261 GBM cells in a transwell coculture system to test the hypothesis that TAMs interact with GBM cells to regulate ferroptosis. We have also used both male and female TAMs to account for the highly sex biased nature of glioblastoma [16, 17]. Moreover, we explore a novel mechanism by which the TAMs regulate ferroptosis involving the release of extracellular vesicles (EVs) containing iron-rich H-Ferritin (FTH1). This TAM-mediated iron release reduces total cellular iron, thereby protecting GBM cells from ferroptosis. This mechanism is mediated by TNF-α secretion from TAMs and is independent of FPN-mediated iron export. Thus, it may be essential for protecting GBM cells from ferroptosis during tumor-induced inflammation. Our results elucidate the potential of combining EV inhibition with ferroptosis inducers for enhanced tumor cell death in GBM.

## Material and methods

### Isolation of primary bone marrow-derived murine macrophages

The isolation of primary macrophages from the bone marrow of C57BL/6J (RRID: IMSR_JAX:000664) mice was preformed using previously reported protocols [18, 19]. Briefly, femurs and tibias were collected from C57BL/6J mice (age 4–6 weeks) and flushed with DMEM with GlutaMAX (ThermoFisher Scientific, Catalog: 61870036) to collect bone marrow. The bone marrow was washed and run through a 70 μm cell strainer and cultured in non-tissue culture-treated Petri dishes in DMEM with GlutaMAX with the addition of 20% v/v conditioned media from L929 (RRID: CVCL_0462) cells and 30% v/v FBS (Macrophage growth media/MGM) to serve as a source of M-CSF for differentiation of bone marrow progenitor cells into macrophages. Additional media was added to the Petri dish on day 3 and day 6. On day 7, the differentiation was confirmed by looking at the cell morphology, adherence to the Petri dish, and expression of macrophage-specific markers (F4/80 and CD11b). The animal experiments were approved by the Institutional Animal Care and Use Committee (IACUC), Penn state College of Medicine.

### Cell culture

GL261 (RRID: CVCL_Y003) cells were obtained from the Developmental Therapeutic Program, NCI. GL261 cells were cultured in DMEM with GlutaMAX (ThermoFisher Scientific, Catalog #: 61870036) with 10% fetal bovine serum (GeminiBio, Catalog number: 100–106), and 1% Penicillin-Streptomycin (ThermoFisher Scientific, Catalog #: 15140-122). Cells were maintained in a humidified tissue culture incubator with 5% CO2 at 37 °C. For experiments, cells up to passage number 8 post-thawing were included to maintain reproducibility. All cell lines tested negative for mycoplasma contamination.

### TAM and GL261 co-culture

For the co-culture experiments, the GBM cells were cultured on a 12-well plate, and TAMs were cultured on transwell inserts of pore size 0.4 μm to facilitate the free exchange of secreted proteins and EVs. After 24 h of coculture, the upper insert containing the macrophages was separated, and the lower chamber containing the GL261 cells was used for downstream analysis and/or further RSL3 treatment.

### RSL3 treatments

RSL3 (Catalog No. S8155, Selleckchem) was used to induce ferroptosis in GBM cells. Briefly, 1 μm or 3 µM (depending on the experiment) of RSL3-containing DMEM media was added to the GBM cells for 24 h to induce ferroptosis. The expression of GPX4 was confirmed by Western blot to verify RSL3 activity. The position of the treatment and control groups on the multiwall plate was chosen by simple randomization.

### Lipid peroxidation analysis

Cells were plated in 12-well tissue culture plates and then treated as indicated. The cells were incubated with 5 µmol/L of C11-BODIPY 581/591 (Image-iT Lipid Peroxidation Kit, C10445, Invitrogen) for 30 min at 37 °C, followed by a single wash with PBS. Lipid ROS generation was assessed by fluorescence microscopy using an ECHO Revolve microscope. The reduced (590 -red) to oxidized (510-green) ratio was calculated to determine the lipid peroxidation.

### Labile iron pool assay

The labile iron pool in the cells was analyzed by staining the cells with BioTracker Far-red Labile Fe^2+^ Dye (Sigma-Aldrich, SCT037). Briefly, the cells were treated with 5 µM of Far-red dye in serum-free media and incubated for 1 h at 37 °C. After staining, the cells were washed once with PBS and detached from the plate using 0.25% trypsin EDTA (Sigma, #T4049). The cells were further centrifuged at 300 g for 5 min and resuspended in fresh PBS. Finally, the cells were filtered through a cell strainer (70 μm nylon mesh) and analyzed using a flow cytometer. The fluorescence signal was recorded using the APC filter.

### Total iron determination

The total iron content of cell lysates was assessed using the iron assay kit (ab83366, Abcam). Samples were prepared according to the manufacturer’s instructions and measured on a plate reader.

### Cell viability assay

Cell viability after RSL3 treatment was analyzed using the CellTiter-Glo^®^ luminescent cell viability assay (Promega), which records luminescence on a plate reader.

### Immunoblot analysis

Protein expressions were detected by immunoblot as previously described [20]. Briefly, cells were lysed using RIPA buffer (Sigma) and protease inhibitor cocktail (PIC, Sigma). Subsequently, total protein was quantified by BCA Protein Assay (Pierce), and equal amounts of protein were loaded onto a 4 to 20% Criterion TGX Precast Protein Gel (Bio-Rad). Proteins were transferred onto PVDF membrane and probed for FTH1 (Cell Signaling Technology, 1:1000, 4393 S), beta-actin (Sigma, 1:1000, A5441), FTL (Abcam, 1:1000, ab69090), CD63 (Invitrogen, 1:250, 1062D), IRP2 (cell Signaling Technology;1:1000, #37135), FPN (alpha diagnostic, 1:1000, MTP11-S), TNF-aplha (Cell Signaling 1:1000, #3707), CD9 (Invitrogen; 1:1000 MA5-31980), CD81 (Invitrogen; 1:1000 MA5-32333), Calnexin (Cell Signaling Technology; 1:1000, 2679), Flotillin 1 (Cell Signaling Technology; 1:1000, 18634), nSMase2 (Sigma, 1:1000 MABC1558). A corresponding secondary antibody conjugated to HRP was used (1:5000, GE Amersham), and the bands were visualized using ECL reagents (PerkinElmer) on an Amersham Imager 600 (GE Amersham). Mouse recombinant TNF-α (Cell Signaling #24095) was used to induce FTH1 release in GL261 cells. The total protein present in the lane was determined by Ponceau Staining and Sypro Ruby staining (SYPRO™ Protein Gel Stains, Invitrogen S12000). CD9 and CD81 immunoblots were performed in non-reducing conditions. The images were quantified with ImageJ software (NIH, Bethesda, MD, USA) and normalized to the respective loading controls.

### ^57^Fe treatment and ICP-MS

The cells were treated with 100 μm ^57^FeCl_3_ (supplemented with 10 μm sodium ascorbate) in serum-free media overnight. Now loaded with isotopically labeled iron, the cells were washed with PBS and cultured in fresh, serum-free media. After 24 h, the cell lysates and the media were collected. The lysates and media were prepared by nitric acid digestion for ICP-MS (Inductively Coupled Plasma Mass Spectrometry) analysis, and the amount of ^57^Fe was quantified. Concentrations of ^57^Fe were normalized to lysate protein concentration to account for slight variations in seeded cell number.

### EVs isolation

The EVs isolation from the cell culture media was completed as described previously in accordance with the MISEV guidelines [21–23]. Briefly, the conditioned media were collected and centrifuged (at 4 °C) at (i) 300 g for 10 min to remove the cell pellets, and (ii) the supernatant was centrifuged at 2000 g for 10 min to remove the dead cell pellet. The resultant supernatant was centrifuged at 4000 g for 30 min to remove the cell debris pellet. The resultant supernatant was collected in a 100 K filter (Amicron Ultra − 15, Centrifugal filters, Merck Millipore Ltd) to concentrate the media. The concentrated media were collected and centrifuged at 100,000 g for 70 min at 4 °C (Beckman coulter, TLA 100.3). The supernatant was discarded, and the pellet was washed with PBS and again centrifuged at 100,000 g for 70 min with the resultant EVs pellet used for downstream processing. The EVs were kept frozen at -20 °C for medium-term storage.

### Transmission electron microscopy

Transmission Electron Microscopy (TEM) was performed as previously described [22]. Briefly, 10 µl of EVs solution was placed on parafilm. Formvar-coated copper grids were then placed on top of the drops and incubated for 20 min. The copper grids were then incubated with a 4% solution of paraformaldehyde in 0.1 M PBS for 20 min, washed thrice with PBS for 1 min each, incubated with 1% glutaraldehyde in 0.1 M PBS for 5 min, washed with distilled water for 2 min, washed thrice with PBS for 2 min each, contrasted with 1% uranyl acetate for 20 s, and then observed by TEM (JEOL-1400).

### Nanoparticle tracking analysis

EVs quantification was performed using NanoSight NS300 (Malvern Instruments Ltd, Malvern). Briefly, EV samples were diluted in 1 ml of particle free-water, and each sample was loaded by syringe pump into the NanoSight NS300 (Malvern Instruments Ltd, Malvern) set in scatter mode, and five 60-s videos were generated at 24.98 frames/sec. The size distribution and concentration of particles were calculated, and images were acquired using NanoSight software, version 3.2 (Malvern Instruments Ltd).

### TNF-α ELISA

TNF-α secreted by the macrophages into the media was quantified using the Mouse TNF-α ELISA Kit (Proteintech, Cat no: KE10002). Briefly, the serum free media containing secreted proteins from BMDMs and TAMs (BMDMs treated with GL261 conditioned media) were collected and centrifuged at 1000 rpm for 5 min with the temperature of 4 °C. The concentration of TNF-α was then measured by ELISA according to protocols from the manufacturer.

### EVs inhibition studies

GW4869 (N, N’-Bis[4-(4,5-dihydro-1 H-imidazol-2-yl) phenyl]-3,3’-p-phenylene-bis-acrylamide dihydrochloride) is the most widely used pharmacological agent to block EVs generation and reduce EVs release by neutral sphingomyelinase (nSMase). The nSMase activity is important for creating the large lipid raft domains involved in EVs shedding, as a result, inhibition of nSMase reduces the release of EVs from the cells. GW4869 was dissolved in DMSO and treated to the cells at a concentration of 30 µM for 24 h to induce inhibition of EVs release. This dose of GW4869 was chosen because this is the highest dose that can be used for 24 h without inducing cytotoxicity [24]. Hepcidin (MedChemExpress HY-P4373) was added to the GBM cells to inhibit FPN expression at a concentration of 4 µM.

### TCGA analysis

The correlation graph between CD68 and GPX4 was generated using the GLIOVIS (https://gliovis.bioinfo.cnio.es) data visualization tools. The adult TCGA GBM_LGG database was used for analysis and Pearson correlation was used for statistics.

### Statistics

All data were expressed as mean ± SD and the statistical analysis was done using GraphPad Prism 9 (RRID: SCR_002798). Students’ unpaired t-tests were used to compare the two groups. One-way ANOVA followed by Tukey’s post hoc analysis test was used to detect statistical significance (*p* < 0.05) between the multiple groups. The significance annotations are * = *P* ≤ 0.05, ** = *P* ≤ 0.01, *** = *P* ≤ 0.001 for all the figures.

## Results

### TAMs protect GBM cells from RSL3-induced ferroptosis

**Fig. 1:**
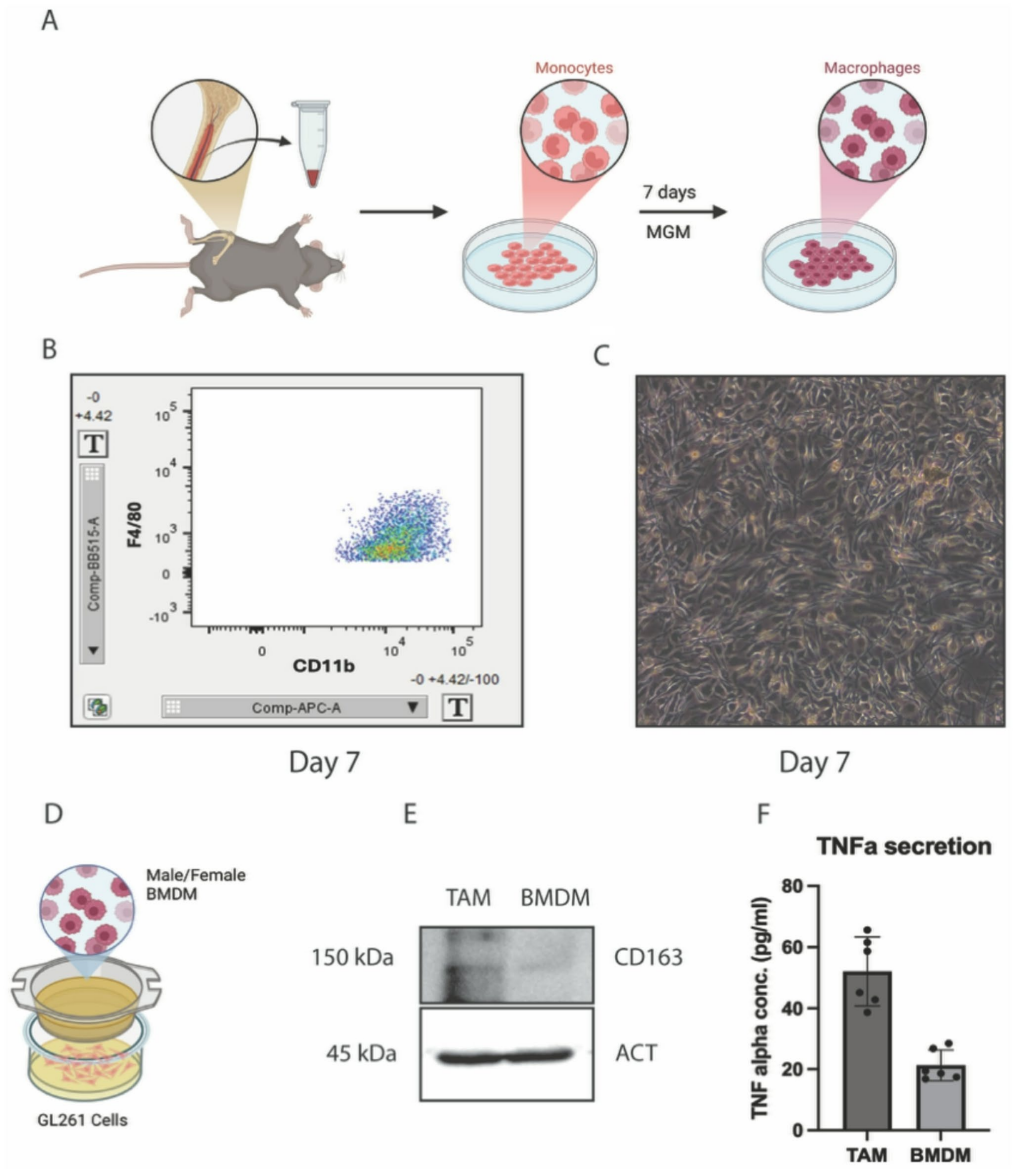
Characterization of BMDM and TAM. **(A)** Graphical representation for BMDM isolation and differentiation. **(B)** Flow cytometry image showing the double positive expression of BMDM markers CD11b and F4/80 on day 7 of differentiation. **(C)** Microscopy image showing the macrophage like morphology of BMDMs on a non-tissue culture treated Petri dish on day 7 of differentiation. **(D)** Graphical representation of the BMDM-GL261 transwell coculture system. **(E)** Immunoblot showing an increase in CD163 expression in TAMs compared with BMDMs. **(F)** ELISA showing increased secretion of TNF-α from TAMs with respect to BMDMs

TAMs have previously been associated with changes in ferroptosis susceptibility in several cancer types [25]. In order to establish our model to study TAM-GBM interaction, we isolated bone marrow cells from C57BL/6 mice and differentiated them to BMDM (Fig. 1A). We further confirmed the differentiation of monocytes to BMDMs by macrophage specific markers (Fig. 1B) and change in morphological phenotypes (Fig. 1C). To interrogate the role of TAMs in regulating the ferroptotic status of GBM cells, we used a transwell coculture system to coculture male and female TAMs with GL261 cells (Fig. 1D). This transwell coculture model to study the interaction between TAMs and cancer cells has been well established in the context of GBM and other tumor types [26, 27]. After 24 h of coculture, the BMDMs are primed by the GL261 cells to become TAMs. We confirmed the TAM phenotype by probing for TAM specific surface marker CD163 expression as well as measuring secretion of TAM associated proinflammatory cytokine TNF-α (Fig. 1E, F).

**Fig. 2:**
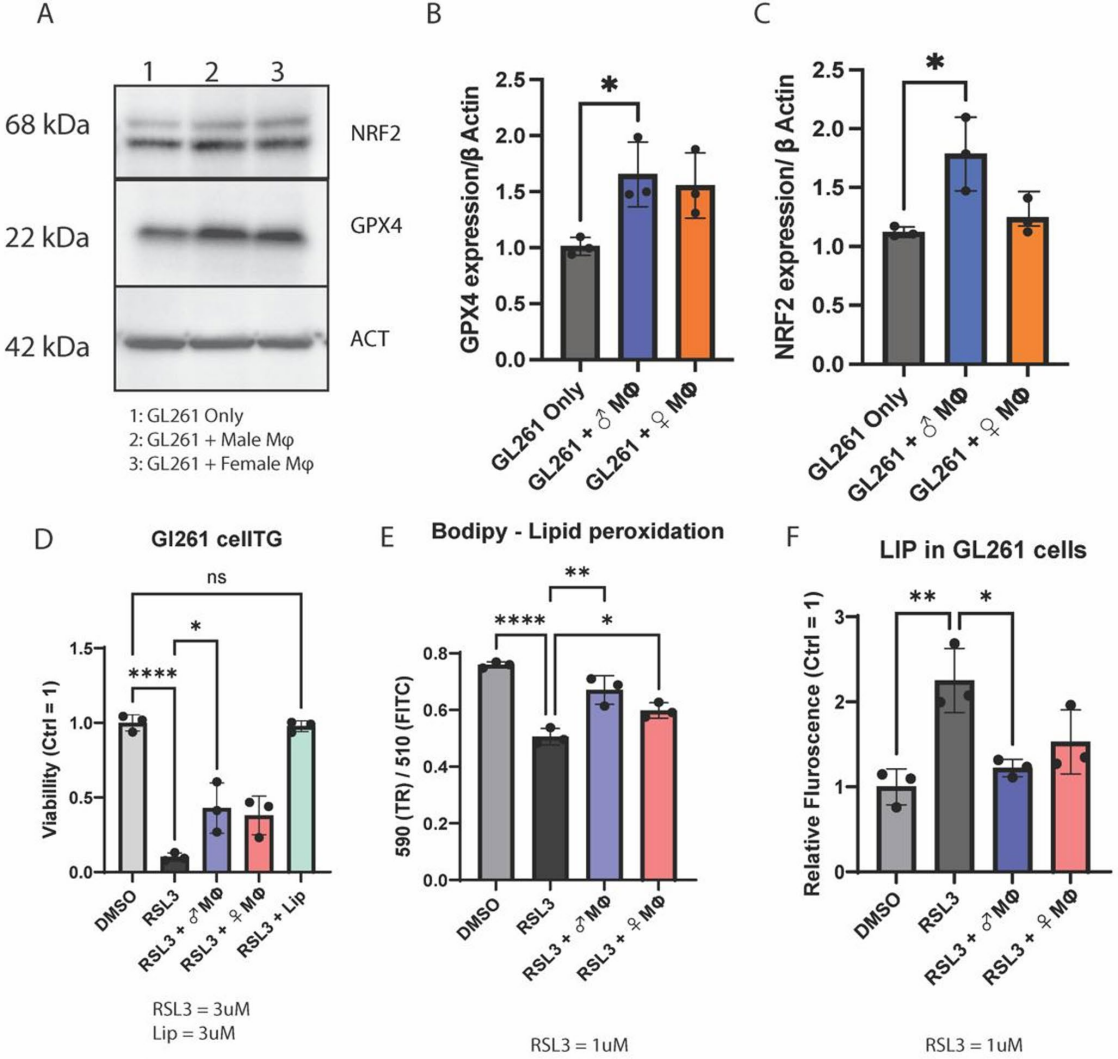
TAMs protect GBM cells from RSL3 induced ferroptosis:

**Fig. 3:**
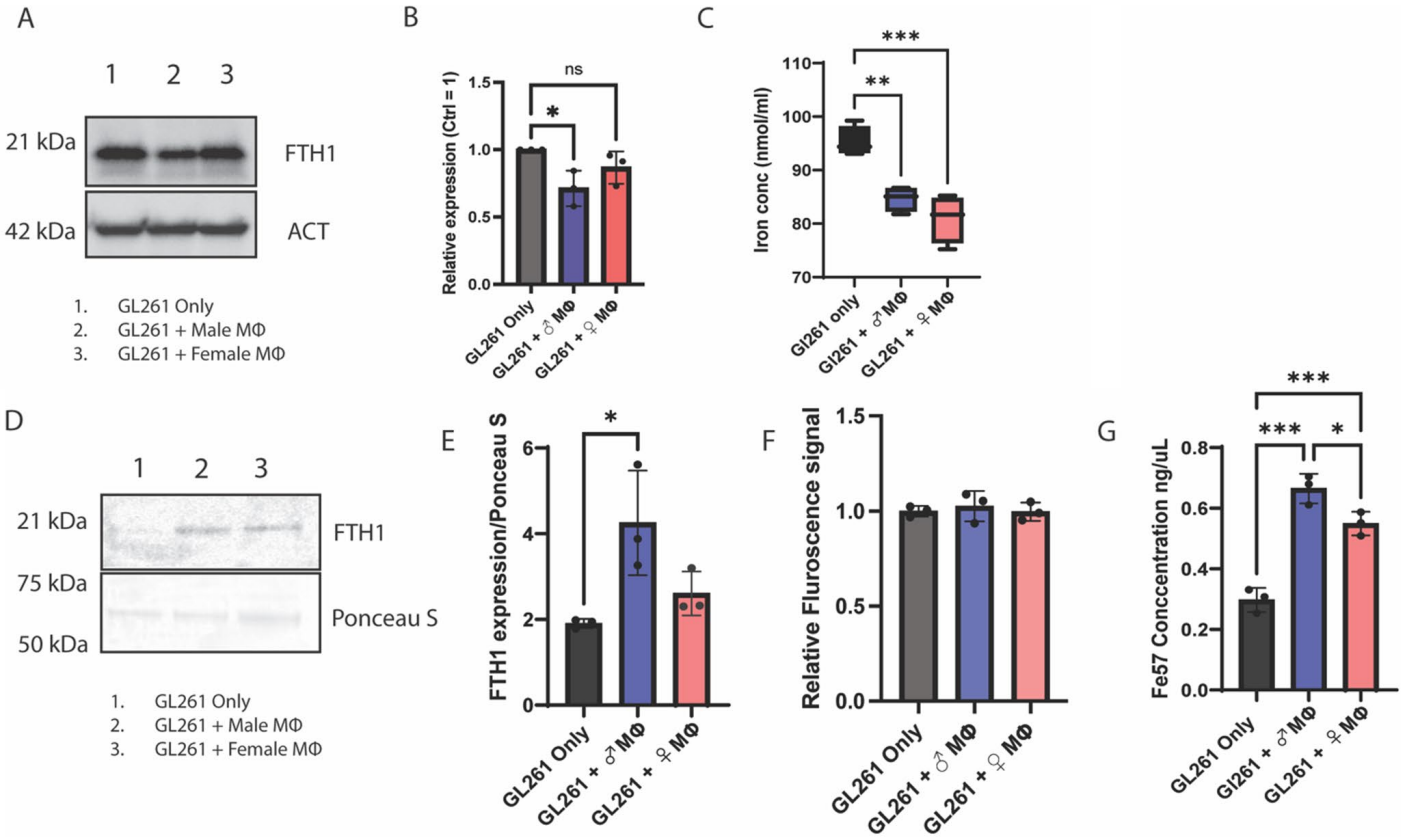
TAMs reduce cellular iron levels in GL261 cells by inducing the release of iron-rich FTH1. **(A)** Immunoblot showing a reduction in cellular FTH1 levels in GL261 cells that were cocultured with TAMs. Beta-Actin is used as loading control. **(B)**: graphical representation of immunoblot data. (*n* = 3, One-way ANOVA). **(C)** Cellular iron measurement assay showing a decrease in total cellular iron (Fe2 + and Fe3+) in GL261 cells after coculture with TAM. (*n* = 3, One-way ANOVA). **(D)** Immunoblot showing higher FTH1 in the secreted media of the GL261 cells that were in coculture with male TAM. Ponceau staining was done to use total protein as a loading control. (**E**) graphical representation of immunoblot data. (*n* = 3, One-way ANOVA). **(F)** Ferro-far red assay showing no significant changes in cellular labile iron pool after coculture with TAM. (*n* = 3, one-way ANOVA). **(G)** Fe57 release from loaded GL261 cells into the coculture media shows that TAMs induce iron release from GBM cells in a sex biassed manner. (*n* = 3, One-way ANOVA)

**Fig. 4:**
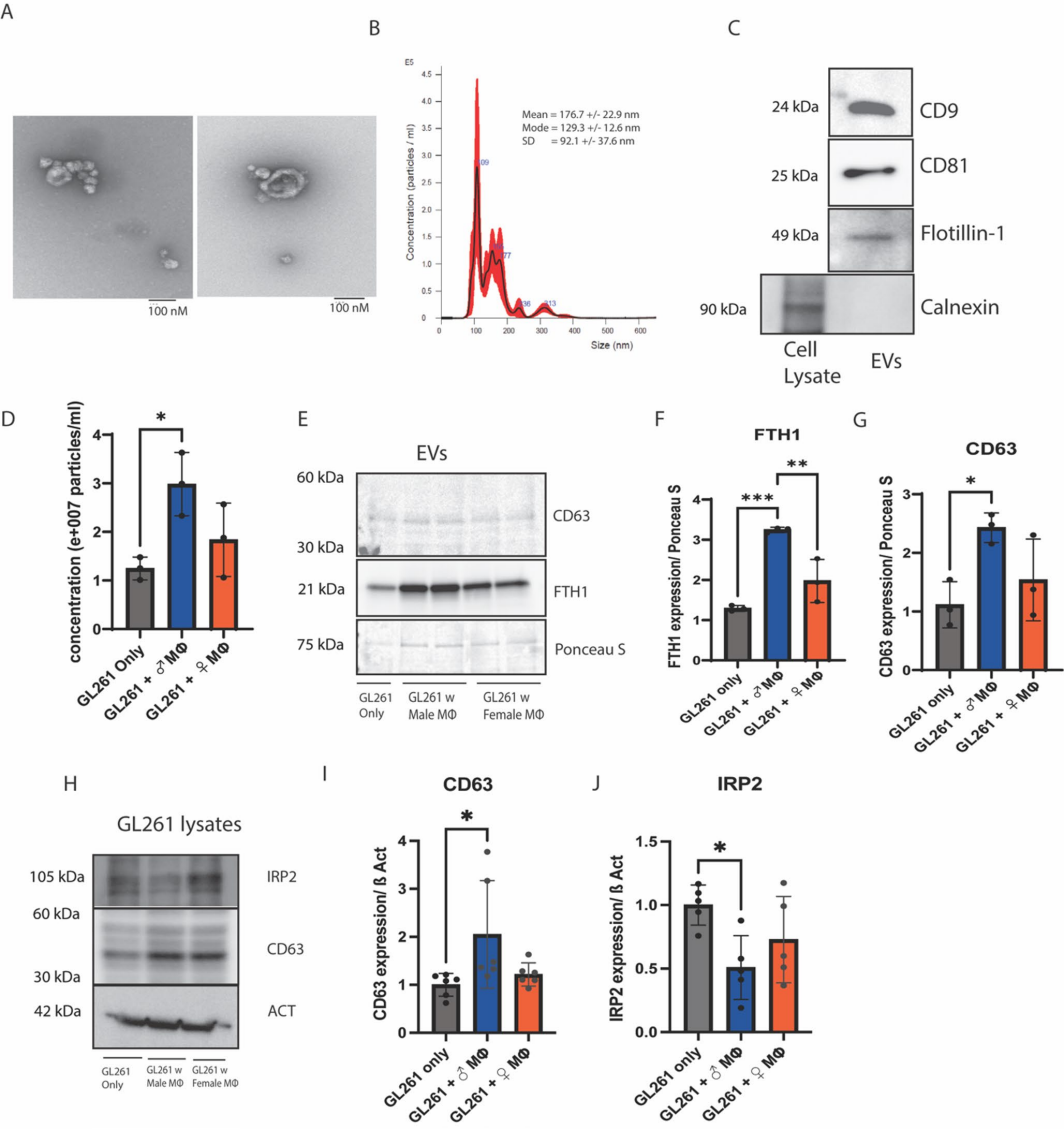
GL261 cells release FTH1 via CD63 positive extracellular vesicles.**(A)** Transmission electron microscopy (TEM) images of isolated EVs from GL261 secreted media. **(B)** Nanoparticle tracking analysis (NTA) analysis showing the size distribution of GL261 secreted EVs. **(C)** Immunoblot showing the expression of EV positive markers (CD9, CD81 and Flotillin-1) and EV negative marker, calnexin which is expressed in GL261 lysates but not in the EVs. **(D)** NTA analysis shows that there is an increase in the concentration of EVs released from GL261 cells after coculture with male TAMs. (*n* = 3, One-way ANOVA). **(E)** FTH1 and CD63 protein expressions in the **EVs** isolated from the GL261 secreted media. The EVs secreted from GL261 cells that were in coculture with male TAMs showed increased CD63 and FTH1 levels. **(F**,** G)** graphical representation of immunoblot data. (*n* = 3, One-way ANOVA). **(H)** Immunoblot showing an increase in cellular CD63 levels in GL261 cells that were cocultured with male TAMs. Immunoblot showing a reduction in cellular IRP2 levels in GL261 cells that were cocultured with male TAMs. Beta-Actin is used as a loading control. **(I)**: graphical representation of CD63 immunoblot data. (*n* = 6, One-way ANOVA). **(J)**: graphical representation of IRP2 immunoblot data. (*n* = 5, One-way ANOVA)

**Fig. 5:**
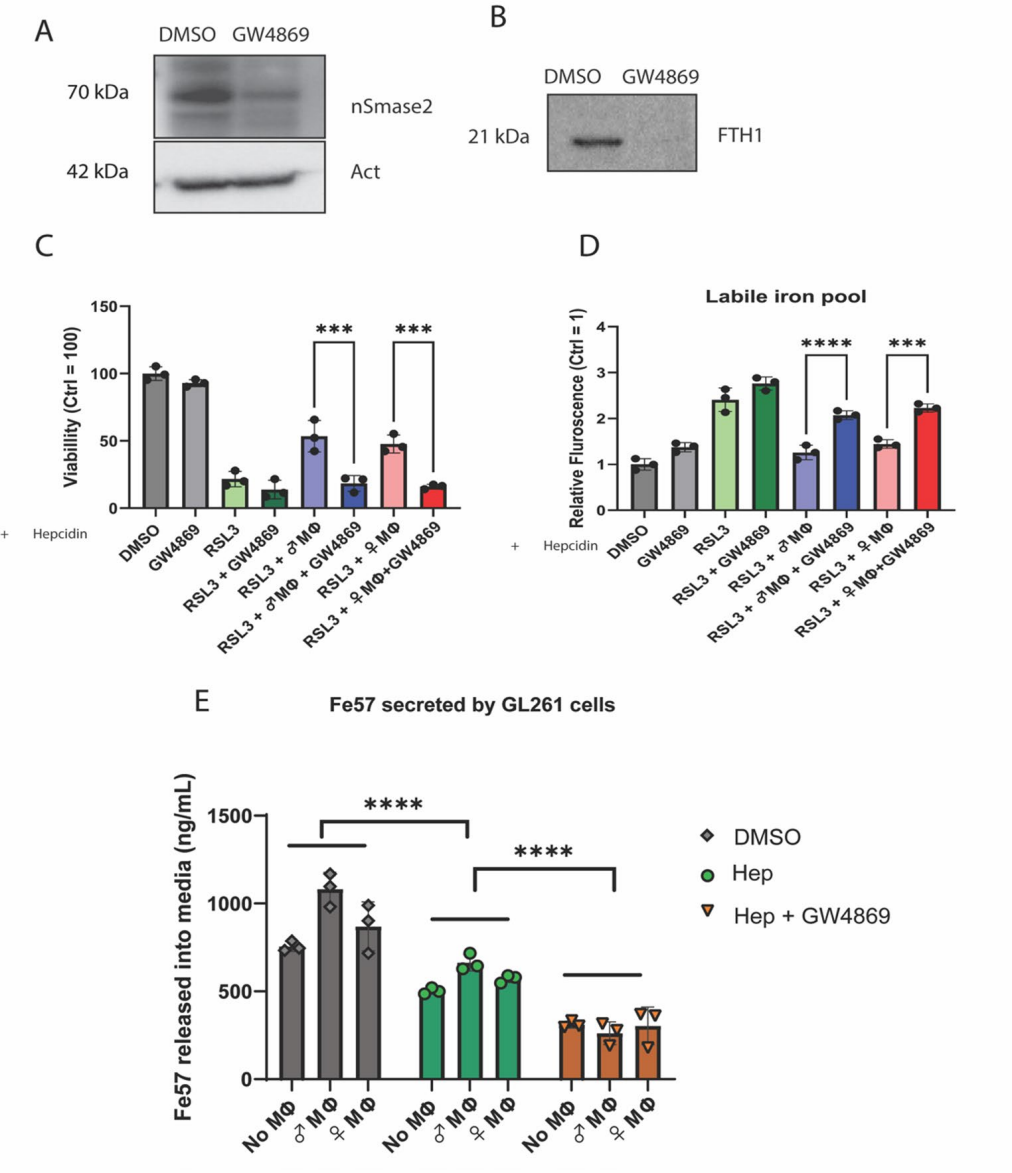
Inhibition of EVs secretion resensitizes GL261 cells cocultured with TAMs to RSL3 induced ferroptosis in the presence of Hepcidin: (**A**) Immunoblot showing inhibition of nSMase2, a key enzyme involved in EVs secretion by GW4869 (30 μm) in GL261 cells. Beta-Actin is used as loading control. (**B**) Immunoblot showing GW4869 (30 μm) treatment caused a decrease in FTH1 release from GL261 cells. (**C**) Cell Titer Glo viability assay showing that in the presence of Hepcidin, GL261 cells in coculture with TAMs become resensitized to RSL3 induced ferroptosis after EV inhibition with GW4869. (RSL3 = 3 μm, Hepcidin = 4 μm)) (n = 3, One-way ANOVA). (**D**) Labile iron pool assay showing that in the presence of Hepcidin, GL261 cells in coculture with TAMs show increased labile iron after EV inhibition with GW4869. (Hepcidin = 4 μm) (n = 3, One-way ANOVA). **(E)**Fe57 release measurement from GL261 cells after treatment with DMSO (control), Hepcidin, and Hepcidin + GW4869. There is a decrease in ^57^Fe release with respect to the control after Hepcidin treatment, and a further decrease in ^57^Fe after Hepcidin + GW4869 treatment. (*n* = 3)

**Fig. 6:**
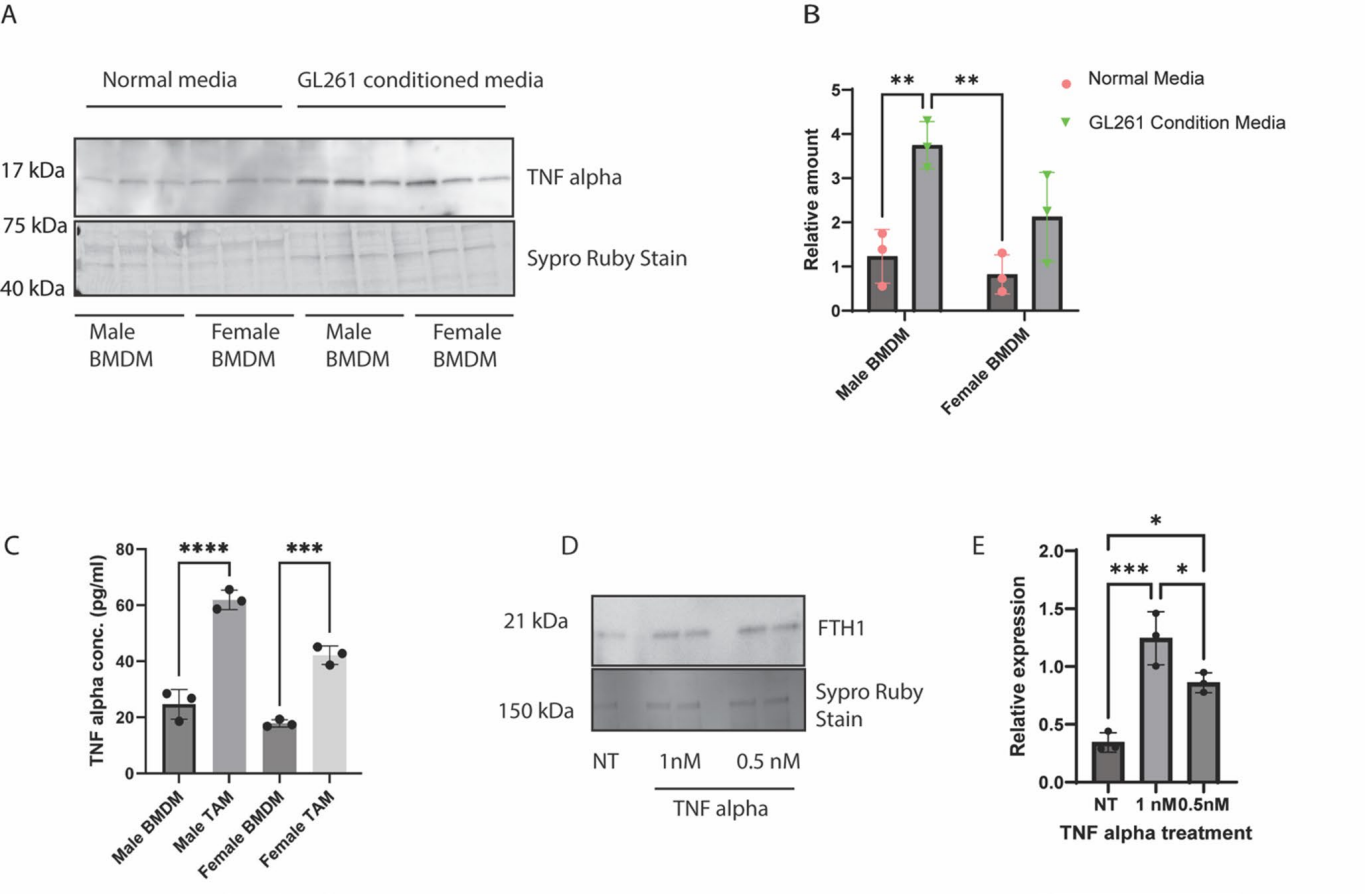
TNF-α signaling acts as a key player in TAM-induced FTH1 release from GBM cells. **(A)** Immunoblot showing BMDMs cultured in the presence of GL261 conditioned media secrete an elevated amount of TNF-α. Sypro ruby staining was done to normalize the total secreted protein. **(B)** graphical representation of immunoblot data. (*n* = 3, two-way ANOVA). (**C**) ELISA showing TAMs (BMDM pretreated with GL261 conditioned media) secrete higher amount of TNF-α than BMDMs (*n* = 3, one way ANOVA). **(D)** Immunoblot showing recombinant TNF-α treatment causes an increase in FTH1 secretion from GL261 cells in a dose-dependent manner. **(E)** graphical representation of immunoblot data. (*n* = 3, One-way ANOVA)

Following coculture, we used immunoblot analysis to study GPX4 and NRF2 expression in GL261 cells, two key proteins regulating ferroptosis. We observed that TAMs induced an increase in GPX4 and NRF2 expression in GL261 cells but only those co-cultured with male TAMs reached statistical significance. (Fig. 2A, B, C). Next, to analyze the impact of TAMs on tumor cell ferroptosis, 3 µM of RSL3 was added to the wells for 24 h to induce ferroptosis. RSL3 induces ferroptotic stress by inactivating glutathione peroxidase 4 (GPX4). After 24 h of treatment, GBM cell viability in the lower chamber was determined using Cell Titer Glo. The assay showed that GBM cells cocultured with macrophages had significantly more viable cells after RSL3 treatment (Fig. 2D). To confirm that cell death was due to ferroptosis, we added liproxstatin-1 (Lip), a potent ferroptosis inhibitor, and observed that Lip restored cell viability after RSL3 treatment (Fig. 2D**).** Lipid peroxidation is a marker of ferroptosis. We investigated lipid peroxidation in GBM cells using C11-BODIPY 581/591 staining. Using a lower dose of RSL3 (1 µM) to induce non-lethal ferroptotic stress, we found a significant decrease in the 590/510 (non-peroxidized/peroxidized lipids) ratio, indicating higher lipid peroxidation in non-cocultured GBM cells treated with RSL3 than control (DMSO). However, GBM cells in coculture showed a significant rescue in the 590/510 ratio, indicating that macrophages reduced lipid peroxidation in GBM cells after RSL3 treatment (Fig. 2E). We used both male and female macrophages to determine potential sex differences. However, despite sex-biased trends in lipid peroxidation, they did not reach significance. Finally, we observed that RSL3 treatment increased the LIP in GL261 cells by measuring ferrous (Fe 2+) iron via BioTracker Far-red Labile Fe^2+^ Dye. (Fig. 2F). The RSL3-treated GL261 cells that were in coculture with macrophages had significantly reduced LIP than those cultured alone. In line with our cell culture observations, we have also seen a positive correlation between GPX4 expression and CD68, a marker for macrophages, in patient GBM tissue transcriptome, suggesting that higher TAM infiltration may contribute to resistance to ferroptosis in GBM tissue (Fig. S1).

### TAMs reduce cellular iron levels in GL261 cells by inducing the release of iron-rich FTH1

Iron metabolism plays a crucial role in regulating ferroptosis [28]. To determine whether TAMs protect GBM cells from ferroptosis by altering their iron levels, we assessed the iron status of GBM cells by quantifying key cellular iron indicators, including FTH1, FPN, the Labile iron pool (Fe2+), and total cellular iron (Fe3 + and Fe2+). We observed a significant decrease in cellular FTH1 in GL261 cells when cocultured with TAMs, with a notable sex bias, where the reduction in FTH1 in cells cocultured with male TAMs was the most pronounced (Fig. 3A, B). Then, we measured the total iron, which includes both the labile iron pool (Fe2+) and protein-bound (Fe3+) iron, where we observed that coculture with TAM significantly reduced the total iron in GL261 cells (Fig. 3C). Next, we checked the expression of FPN via immunoblotting and observed no alterations in FPN expression in GL261 cells after coculture with male and female TAMs (Fig. S2). To understand how the GBM cells in coculture with TAMs had reduced cellular ferritin and total iron, we hypothesized that TAMs induce the release of iron-bound ferritin from GL261 cells. To address this hypothesis, we cocultured the GL261 cells with male or female TAMs for 24 h. Then, we removed the upper insert containing macrophages and added fresh serum-free media to the GL261 cells in the lower chamber. Next, we collected the media from the TAM-primed GL261 cells after 24 h. Immunoblotting showed that GL261 cells in coculture with TAMs had increased FTH1 secretion in the media but only the increase in FTH1 secretion from GL261 cells exposed to male TAMS reached statistical significance (Fig. 3D, E). However, we did not see any difference in the cellular labile iron pool in cocultured GBM cells or GBM cells grown separately (Fig. 3F). Next, to determine whether FTH1 secretion correlated with iron release, we used ^57^Fe-loaded GL261 cells and repeated the experiment. We observed that the ^57^Fe secretion was significantly increased in TAM-cocultured GL261 cells compared to GL261 cells cultured alone, with a clear male bias where male TAMs induced a higher iron release in GL261 cells than female TAMs (Fig. 3G).

### GL261 cells release FTH1 via CD63 positive extracellular vesicles

We, along with other groups, have previously shown that EVs can mediate cellular secretion of ferritin from endothelial cells and different cancer cells [11, 22]. To interrogate whether TAMs induce FTH1 release from GL261 cells via EVs, we used ultracentrifugation to isolate the EVs secreted by GL261 cells. We confirmed the EVs by transmission electron microscopy (TEM) (Fig. 4A) and nanoparticle tracking analysis (NTA) (Fig. 4B). Both of these methods are considered the gold standard for the characterization of EVs. We also confirmed the EVs by checking the expression of the EV-specific positive markers CD9, CD81, Flotillin-1 and negative marker Calnexin (Fig. 4C). Next, we used NTA to determine the concentration of EVs released and found that TAMs induced increased concentration of EV secretion which was male-biased (Fig. 4D). We interrogated the EVs secreted from GL261 cells by immunoblot and observed that the EVs secreted from GL261 cells cocultured with TAMs had increased FTH1 and CD63 protein levels compared to non-cocultured GL261 cells with a male bias (Fig. 4E, F, G). Furthermore, to understand the mechanism regulating EV release, we used immunoblotting to examine the cellular expression of Iron Regulatory Protein 2 (IRP2) in GBM cells cultured alone or in coculture with TAMs. We observed that GBM cells cocultured with male TAMs had a significant reduction in cellular IRP2 (Fig. 4H, J). The IRP2 expression in GBM cells cocultured with female tumor-associated macrophages (TAMs) showed a lower trend compared to GBM cells alone. IRP2 impacts cellular iron uptake and release by post-transcriptionally regulating the expression of several key proteins involved in iron homeostasis. One such protein is CD63, which is involved in the biogenesis of EVs and is a prominent EV marker. Thus, we checked the cellular expression of CD63 in the GBM cells. We observed that GL261 cells co-cultured with male TAMs had an increased expression of cellular CD63. (Fig. 4H, I). Additionally, as shown in Fig. 3(A, B) we have already observed that coculture with TAMs causes a reduction in FTH1 levels in GL261 cells. Taken together TAMs induce decrease in cellular IRP2 and FTH1 and increase in cellular CD63 in GL261 cells.

### Pharmacological inhibition of EVs secretion resensitizes GL261 cells cocultured with TAMs to RSL3 induced ferroptosis in the presence of Hepcidin

EV-mediated iron release is a non-canonical form of cellular iron release, which is not regulated by the Hepcidin/FPN axis. Hepcidin is a protein, elevated during inflammation, that inhibits cellular iron release by degrading the iron exporter FPN. To understand whether the EVs-mediated FTH1 release pathway is potentially involved in ferroptosis resistance during inflammation, we treated the co-culture with Hepcidin, a protein that is elevated during inflammation. Hepcidin reduces the amount of cellular FPN (Fig. S3), and it blocks FPN-mediated iron release. GW4869 is a sphingomyelinase inhibitor that reduces EVs biogenesis by decreasing nSMase2 (Fig. 5A). Treatment with GW4869 decreased the FTH1 release from GL261 cells (Fig. 5B). To confirm that the suppression of ferroptosis is regulated by EVs release during inflammation, we treated the GL261 cells with GW4869 in the presence of Hepcidin (4 μm) to maximally inhibit iron export from the cell. This treatment resulted in the loss of ferroptotic resistance in TAM-cocultured GL261 cells after lethal dose of RSL3 (3 μm) treatment (Fig. 5C). Further we interrogated the labile iron (Fe2+) levels in GL261 cells after lower dose of RSL3 treatment (1 μm). We observed that under RSL3 induced ferroptotic stress during high hepcidin conditions, blocking EVs release resulted in higher LIP in GL261 that were in coculture with TAMs (Fig. 5D). Finally, we loaded the GL261 cells with ^57^Fe (100 μm) and measured iron release after Hepcidin and Hepcidin + GW4869 treatment. Hepcidin treatment significantly decreased iron release compared to control. The greatest decrease in iron release was obtained when Hepcidin and GW4869 treatments were combined (Fig. 5E).

### TNF-α signaling acts as a key player in TAM-induced FTH1 release from GBM cells

TAM-mediated changes in the GBM cells are normally carried out by cytokines that are secreted from the macrophages, which interact with the tumor cells to induce downstream signaling. Previous research from our laboratory and others has shown that the proinflammatory cytokine TNF-α can induce FTH1 release in different cell types, such as endothelial cells and macrophages [21, 29]. To determine whether TAMs have an elevated secretion of TNF-α, we treated the BMDMs with GL261 conditioned-media for 24 h. Next, we replaced the old media with fresh serum-free media for 24 h and checked the levels of TNF-α in the media by immunoblot. Immunoblot analysis showed that the BMDMs treated with GL261-conditioned media secreted significantly higher amounts of TNF-α, with a strong bias towards male TAMs (Fig. 6A, B). Subsequently, we used ELISA to determine the concentration of secreted TNF-α from the macrophages and observed that male TAMs (BMDMs pre-treated with GL261 conditioned media) showed a three-fold increase in TNF-α secretion (from 23pg/ml to 63pg/ml) compared to male BMDMs, whereas it was two-fold increase in females (18pg/ml to 40pg/ml) (Fig. 6C). Next, we treated recombinant TNF-α to GL261 cells and observed that TNF-α treatment induced an increase in FTH1 release from the GL261 cells in a dose-dependent manner via immunoblot analysis (Fig. 6D, E). The above data indicate that TNF-α is one of the key factors that causes TAM-induced FTH1 release from GL261 cells.

## Discussion

Ferroptosis is an intracellular iron-dependent form of cell death involving lipid peroxidation and membrane instability. Multiple studies have shown that tumor cell populations that are highly metastatic or resistant to anticancer drugs show increased sensitivity to ferroptosis [30, 31]. Hence, understanding the regulation of ferroptosis in cancer cells can provide novel approaches to cancer therapy [32]. Susceptibility to ferroptosis can depend on intrinsic factors such as lipid metabolism, iron homeostasis, and antioxidant defense, as well as extrinsic factors such as other cells in the tumor microenvironment, including non-tumor cells and infiltrating immune cells. However, studies on cancer ferroptosis that consider both intrinsic factors and extrinsic interactions in the TME are scarce [33].

TAMs are the most abundant non-tumor cell type in the GBM microenvironment. They are essential for maintaining iron homeostasis in the TME and thus may affect the sensitivity of GBM cells to ferroptosis. Previous studies in prostate and cervical cancer have shown that TAMs can affect tumor ferroptosis by altering lipid metabolism [34, 35]. However, in GBM, there have been no conclusive studies exploring the role of TAMs in regulating ferroptosis.

In this current study, we demonstrated that TAMs make GBM cells less susceptible to ferroptosis. Coculture with TAMs increased GPX4 and NRF2 expression in GL261 cells in a male-biased manner. Both proteins are involved in the antioxidant pathway, and their upregulation indicates that TAMs can affect the redox status of GBM cells. Our results support previous studies where TAMs have been reported to increase NRF2 expression in cancer cells [36]. TAMs conferred significant protection to GL261 cells from ferroptosis after RSL3 treatment. We have used multiple approaches to characterize ferroptosis in GL261 cells, in line with the current guidelines for ferroptosis detection in cell culture systems [37] Our data support previous studies in other cancers where TAMs have been shown to protect cancer cells from ferroptotic stress [25, 38].

Cellular iron status plays a central role in regulating ferroptosis. Considering this, we hypothesized that TAMs could alter the iron status of GBM cells, which resulted in making them more resistant to ferroptosis. TAMs decreased the FTH1 and total iron levels in GL261 cells in a sex biased manner. However, TAMs didn’t affect FPN expression in GL261 cells. FTH1 has been shown to act as an iron delivery protein that can be involved in iron translocation from one cell to another [22, 39]. We observed increased FTH1 secretion from GL261 cells after coculture with TAMs. ^57^Fe experiments further confirmed that the FTH1 were iron rich in nature. These data indicate that TAMs induce FTH1-bound iron release from GBM cells, representing a major pathway for iron release.

Previous studies have indicated that cells can release iron via multi-vesicular body (MVB)/exosome trafficking of iron-rich ferritin [11, 22]. Hence, we hypothesized that GL261 cells release FTH1-bound iron via EVs. EVs isolation and further characterization indicated that TAMs induced increased FTH1 levels in the GL261-secreted EVs. For our studies, we have used the differential ultracentrifugation method, which is the gold standard for EVs isolation as it provides good specificity without sacrificing the recovery [23, 40]. Furthermore, TAMs induced an increase in secreted EVs concentration. EV biogenesis is a tightly regulated process. Previous studies have shown that intracellular proteins involved in iron sensing, called Iron Regulatory Proteins (IRPs), regulate EVs biogenesis by regulating the expression of CD63 [22, 41]. In our system, we observed that TAMs cause a decrease in cellular IRP2 levels and an increase in cellular CD63 levels in GL261 cells in a male-biased manner. IRP2 can regulate the translation of CD63 mRNA by binding to the IRE sequence in the 5’ UTR region. In the absence of IRP2, the IRE in the 5’ UTR of CD63 mRNA becomes unbound, resulting in increased synthesis of CD63 protein and EVs biogenesis. Our results indicate that TAMs induce increased CD63-positive EVs secretion via decreasing the expression of IRP2 in the GL261 cells. These secreted EVs play a pivotal role in cell-cell communication in the TME. EVs can carry signaling molecules that can be delivered to other tumor and non-tumor cells in the TME and initiate a signaling cascade. Previous reports have indicated the role of tumor secreted EVs in TAM polarization, immune infiltration and TME organization [42]. Recent studies have showed that EVs can also play a role in exocytosis to release specific proteins out of the cell into the extracellular matrix [41, 43].

There are two major ways of cellular iron release. The first one involves the release of ferrous iron by the only iron exporter present in cells, FPN. This is the canonical mechanism for cellular iron release and has been studied extensively in the context of cellular iron homeostasis and regulation of ferroptosis [14, 15]. The second pathway involves the release of protein-bound iron via EVs, which is becoming a more greatly appreciated significant pathway. Both pathways remain active in cancer cells and can contribute to ferroptosis resistance by reducing cellular iron. However, several tumors are characterized by a proinflammatory environment. Inflammation increases the expression of Hepcidin in the TME. Hepcidin is a peptide hormone that binds to and degrades FPN, thereby blocking FPN-mediated iron release in a proinflammatory tumor [15]. During this scenario, the noncanonical pathway of EVs-mediated iron release can work as the only functional mechanism by which cancer cells get rid of cellular iron to resist ferroptotic stress. Our data show that TAMs don’t alter the expression of FPN or the amount of ferrous iron in GL261 cells, indicating no changes in the canonical FPN-mediated iron release. Instead, TAMs utilize the alternate IRP2-regulated (MVB)/exosome trafficking pathway to secrete iron-rich FTH1. This indicates that TAMs could suppress ferroptosis by this pathway in a proinflammatory tumor microenvironment. To test this, we first treated the GL261 cells with hepcidin in the coculture model and then blocked the EVs release by GW4869, an inhibitor of EVs biogenesis. Treating the GL261 cells with GW4869 in the presence of Hepcidin, resensitized the TAM cocultured cells to lethal doses of RSL3 induced ferroptosis. This corresponded with higher LIP levels in EV-inhibited TAM cocultured GL261 cells compared with the non-EV-inhibited cells under non-lethal doses of RSL3. These results indicate that EVs-mediated FTH1-bound iron release is the major pathway of iron release by GL261 cells during proinflammatory conditions, and TAMs utilize this pathway to confer ferroptotic resistance to GBM cells. Blocking both the iron release pathways resensitized the GL261 cells to ferroptosis, indicating the potential of combining EVs inhibition drugs with ferroptosis inducers as an effective way to target GBM cells.

Previous studies have shown that TNF-α, a proinflammatory cytokine, can increase FTH1 release by degrading IRP2 [29]. Our data show that TAMs secrete higher amounts of TNF-α than normal BMDMs, with a moderate male bias. Treatment of recombinant TNF-α to GL261 cells resulted in increased FTH1 secretion from GL261 cells. Our data align with previous work from our laboratory and others, which describes the role of TNF-α in inducing FTH1 release [21, 29].

In summary, this study reveals that iron-rich FTH1 release via CD63-positive EVs is a major mechanism by which TAMs enable GBM cells to develop ferroptotic resistance. The fate of these secreted EVs from the GBM cells cannot be discerned from the current study but are likely taken up by other cells in the TME and even outside the tumor. The role of these secreted EVs in reprogramming the TME or in recruitment of non-cancer cells to the tumor is interesting but speculative at this time. An important limitation of our study is the exclusive use of GL261 cells, which does not capture the intra- and inter-tumoral heterogeneity characteristic of GBM, but our study establishes the foundational framework that TAMs can directly bestow ferroptotic resistance on GBM cells. There appears to be a moderate sex bias favoring males, partially driven by sex biases in TNF-α secretion from TAMs. GBM is a sexually dimorphic disease, with differences in incidence, phenotype, response to therapy, and overall outcome [17]. The male-biased sex differences observed in this cell culture-based study align with the sexual dimorphism that is observed in clinical data and may provide insights into why male GBM is more resistant to standard therapy-induced cell death. Finally, our study suggests the therapeutic potential of using ferroptosis inducers in conjunction with EVs inhibitors in glioblastoma for a more pronounced effect.

## Supplementary Information

Below is the link to the electronic supplementary material.

Supplementary Material 1

## Data Availability

All data generated in this study are available upon request from the corresponding author, Dr. James Connor (mailto: jconnor@pennstatehealth.psu.edu). No datasets were generated during the current study.
