# Supplementary figures and images for: Tumor-associated macrophages promote ferroptosis resistance in glioblastoma by stimulating iron-loaded extracellular vesicle release
Source: J Neurooncol. 2026 Apr 13;177(2):99. doi: 10.1007/s11060-026-05556-w (PMC13076414; doi:10.1007/s11060-026-05556-w)

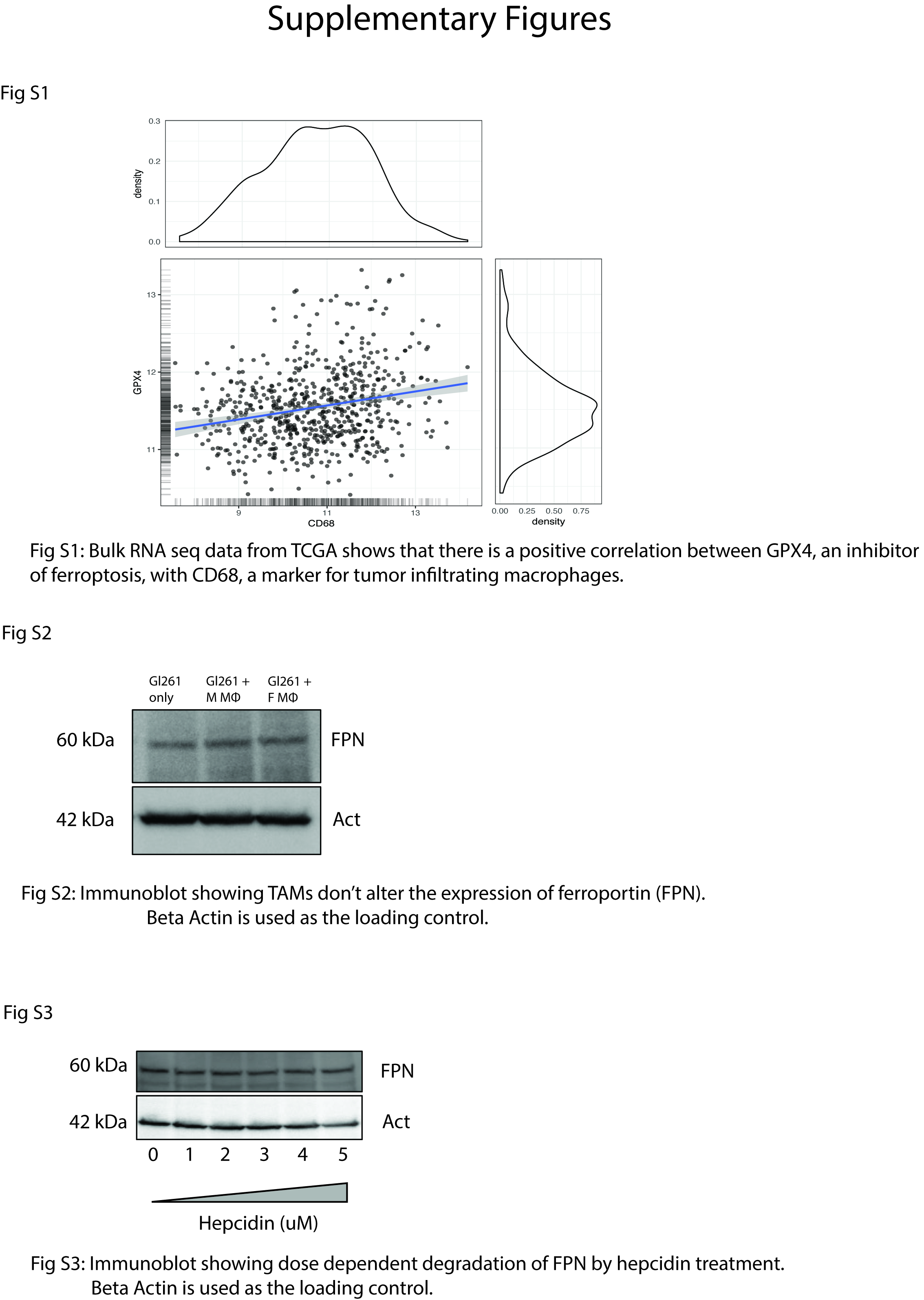

Supplement: Supplementary file 1 — Supplementary Material 1 [file 11060_2026_5556_MOESM1_ESM.tif]
